# Targeting MAPK Pathways by Naringenin Modulates Microglia M1/M2 Polarization in Lipopolysaccharide-Stimulated Cultures

**DOI:** 10.3389/fncel.2018.00531

**Published:** 2019-01-11

**Authors:** Bei Zhang, Yi-Zheng Wei, Guo-Qing Wang, Dai-Di Li, Jing-Shan Shi, Feng Zhang

**Affiliations:** Key Laboratory of Basic Pharmacology of Ministry of Education, Joint International Research Laboratory of Ethnomedicine of Ministry of Education, Zunyi Medical University, Zunyi, China

**Keywords:** neuroinflammation, microglia polarization, lipopolysaccharide, naringenin, MAPK signaling

## Abstract

Neuroinflammation is considered to be an important and inevitable pathological process associated with all types of damages to, and disorders of, the central nervous system. The hallmark of neuroinflammation is the microglia activation. In response to different micro-environmental disturbances, microglia could polarize into either an M1 pro-inflammatory phenotype, exacerbating neurotoxicity, or an M2 anti-inflammatory phenotype, exerting neuroprotection. Therefore, shifting the polarization of microglia toward the M2 phenotype could possess a more viable strategy for the neuroinflammatory disorders treatment. Naringenin (NAR) is naturally a grapefruit flavonoid and possesses various kinds of pharmacological activities, such as anti-inflammatory and neuroprotective activities. In the present study, we aimed to investigate the potential effects of NAR on microglial M1/M2 polarization and further reveal the underlying mechanisms of actions. First, NAR inhibited lipopolysaccharide (LPS)-induced microglial activation. Then, NAR shifted the M1 pro-inflammatory microglia phenotype to the M2 anti-inflammatory M2 microglia state as demonstrated by the decreased expression of M1 markers (i.e., inducible TNF-α and IL-1β) and the elevated expression of M2 markers (i.e., arginase 1, IL-4, and IL-10). In addition, the effects of NAR on microglial polarization were dependent on MAPK signaling, particularly JNK inactivation, as evidenced by the fact that the selective activator of JNK abolished NAR-promoted M2 polarization and further NAR-inhibited microglial activation. Together, this study demonstrated that NAR promoted microglia M1/M2 polarization, thus conferring anti-neuroinflammatory effects via the inhibition of MAPK signaling activation. These findings might provide new alternative avenues for neuroinflammation-related disorders treatment.

## Introduction

Accumulating evidence has confirmed that central nervous system (CNS) is an immunologically privileged site due to the limited inflammatory capacity with the presence of blood-brain barrier and the lack of lymphatic infiltration (Hanisch and Kettenmann, 2007). Recently, neuroinflammation is considered to be an important and inevitable pathological process associated with all types of damages to, and disorders of, the CNS (Gemma, 2010). As the major cellular elements of neuroinflammation, microglia execute specific immune functions to maintain physiological homoeostasis (Dhama et al., 2015). In response to the pathogenic insult to the CNS, microglia become activated and undergo morphological changes with hypertrophy as well as functional transformations (Ji et al., 2013; Huo et al., 2018). Several lines of studies have indicated that microglia activation plays a pivotal role in the pathogenesis of neurological disorders, including trauma, brain infections coma stroke, ischemia, and neurodegenerative diseases (Nimmo and Vink, 2009). Furthermore, the activated microglia consist of two cell populations which have distinct and even opposing functions. These two microglial polarization extremes are termed as the classically activated M1 (pro-inflammatory) and alternatively activated M2 (anti-inflammatory) phenotypes (Hu et al., 2015). In addition, the two microglial distinct functional polarization states were discerned in the neurodegenerative diseases, such as Parkinson’s disease (Bok et al., 2018). Generally, the activated M1 phenotype microglia are characterized by the increased production of pro-inflammatory factors, including tumor necrosis factor-α (TNF-α), interleukin-1β (IL-1β) and the upregulation of inducible nitric oxide synthase (iNOS), CD16, and CD68 (David and Kroner, 2011). Conversely, the activated M2 state microglia are demonstrated to upregulate anti-inflammatory mediators, such as arginase-1 (Arg-1), CD206, and transforming growth factor-β (TGF-β) (Saijo and Glass, 2011). Functionally, the microglia M1 phenotype exacerbates neuronal damage and impedes cellular repair during CNS trauma and disorders. On the contrary, the M2 microglia exert neuroprotection and promote neuronal recovery and remodeling (Mantovani et al., 2013). Therefore, shifting the polarization of microglia toward the M2 phenotype could possess a more viable strategy for the neuroinflammatory disorders treatment.

Naringenin (NAR) is naturally a grapefruit flavonoid and possesses various kinds of pharmacological activities, such as anti-oxidant, anti-inflammatory, cardioprotective and anti-tumor activities (Al-Dosari et al., 2017). The anti-inflammatory effects of NAR are well verified in several different models. NAR protected against airway remodeling after mycoplasma pneumoniae infection via the inhibition of autophagy-mediated lung inflammation and fibrosis (Lin et al., 2018). Also, NAR suppressed the development of precancerous lesions through controlling hyperproliferation and inflammation in the colon of rats (Rehman et al., 2018). Recently, in addition to these beneficial actions, growing interests have been focused on its neuroprotective actions (Kara et al., 2014). First, NAR conferred neuroprotection in Parkinson’s disease animal models and attenuated neuroinflammatory reactions (Magalingam et al., 2015). Moreover, NAR produced analgesic effects via inhibiting oxidative stress and oxidation cytokine production (Manchope et al., 2016). However, whether NAR could promote microglial polarization to M2 phenotype and the mechanisms underlying NAR-mediated anti-neuroinflammatory effects remain unilluminated.

In the present study, we aimed to investigate the potential effects of NAR on microglial M1/M2 polarization and further reveal the underlying mechanisms of actions. Specifically, these findings might provide new alternative avenues for neuroinflammation-related disorders treatment.

## Materials and Methods

### Reagents

Naringenin, lipopolysaccharide (LPS) and Anisomycin (ANI) were purchased from Sigma-Aldrich (St. Louis, MO, United States). Enzyme-linked Immunosorbent Assay (ELISA) kits were obtained from R&D Systems (Minneapolis, MN, United States). The fluorescence probe dichlorodihydrofluorescein diacetate (DCFH-DA) were bought from Sangon Biotech (San Diego, CA, United States). SYBR green polymerase chain reaction (PCR) master mix was purchased from Bio-Rad (CA, United States). Anti-mitogen-activated protein kinase (MAPK) signaling pathway antibodies were purchased from Cell signaling Technology (Beverly, MA, United States). Anti-ionize calcium binding adapter molecule 1 (Iba-1), anti-TNF-α, anti-Arg-1, and anti-β-actin antibodies were obtained from Proteintech Group (Chicago, IL, United States).

### Cell Culture and Treatment

BV-2 cells, an immortalized murine microglial cell line, were obtained from Wuhan University Cell Library (Wuhan, China). Cells were cultured in DMEM/F12 medium with 10% FBS and 1% penicillin/streptomycin at 37°C in a humidified atmosphere containing 95% air and 5% CO_2_. Cells were seeded into 24-well plates at 5 × 10^5^/well or 96-well plates at 1 × 10^5^/well. Cultures were pretreated with NAR (50 μM) for 1 h, and then incubated with or without ANI (5 nM) for another 1 h. Finally, LPS (100 ng/ml) were added to cultures for 24 h. The corresponding indexes were tested.

### MTT Assay

Cells were seeded into 96-well plates at 1 × 10^5^/well. MTT (5 mg/ml) solution was added to each well and continued incubation for 4 h at 37°C. After removing the upper medium, dimethyl sulfoxide (DMSO) was added to each well at 37°C to solubilize formazon. Absorbance values were measured at 490 nm.

### ELISA

Cells were seeded into 24-well plates at 5 × 10^5^/well. The TNF-α and IL-10 levels in the supernatant were detected using ELISA kit according to the manufacturer’s instructions.

### Real-Time RT-PCR Assay

Total RNA was extracted with Trizol agent and purified with RNeasy kit. Iba-1, TNF-α, IL-1β, Arg-1, IL-10, and β-actin genes were amplified using the forward and reverse primers. Real-time PCR was performed using a SYBR Green Supermix according to the instruction and then determined on a CFX96 real-time PCR detection system (Bio-Rad, CA, United States). The target gene expression levels were normalized with that of β-actin using the data analysis software provided with the system.

### Western Blotting

Cells were lysed in RIPA buffer and the lysates were incubated on ice for 15 min and then centrifuged at 12,000 × g for 15 min at 4°C. The protein concentrations were quantified by BCA assay. A total of 10 μg protein were resolved on 10% Bis-Tris Nu-PAGE gels and transferred to the polyvinylidene difluoride (PVDF) membranes and blocked in 5% fat-free milk at room temperature for 2 h. Membranes were incubated with following primary antibodies: anti-phosphorylated c-Jun Nterminal kinase (p-JNK,1:1000), anti-JNK (1:1000), anti-phosphorylated extracellular signal-regulated kinase1/2 (p-ERK1/2,1:2000), anti-ERK1/2 (1:1000), anti-phosphorylated p38 (p-p38,1:1000), anti-p38 (1:1000), anti-Iba-1 (1:1000), and anti-β-actin (1:2000). The membranes were then incubated with horseradish peroxidase (HRP)-conjugated secondary antibodies at 1:2000 for 1 h. The proteins were detected with ECL substrate.

### Immunocytochemical Staining

BV-2 cells were fixed with 4% paraformaldehyde for 30 min at room temperature followed by permeabilization using 0.3% Triton X-100 for 15 min. Cells were then blocked with goat serum at 37°C for 40 min. Cells were concentrated with anti-Iba-1 (1:300), anti-TNF-α (1:300) and anti-Arg-1 (1:300) at 4°C overnight, respectively. The next day, cells were incubated with anti-rabbit-IgG (1: 1500) or anti-mouse-IgG (1: 1500) at 37°C for 1 h. Subsequently, cells were incubated with DAPI for 2 min at room temperature.

### Statistical Analysis

Data were presented as mean ± standard error of the mean (SEM). Statistical significance was analyzed by one-way ANOVA through the GraphPad Prism software (GraphPad Software Inc., San Diego, CA, United States). After ANOVA demonstrated the significant differences, pairwise comparisons between means were accessed by Bonferroni’s *post hoc* tests with correction. *p* < 0.05 was considered as statistically significant.

## Results

### NAR Attenuated LPS-Induced Microglial Activation

**FIGURE 1:**
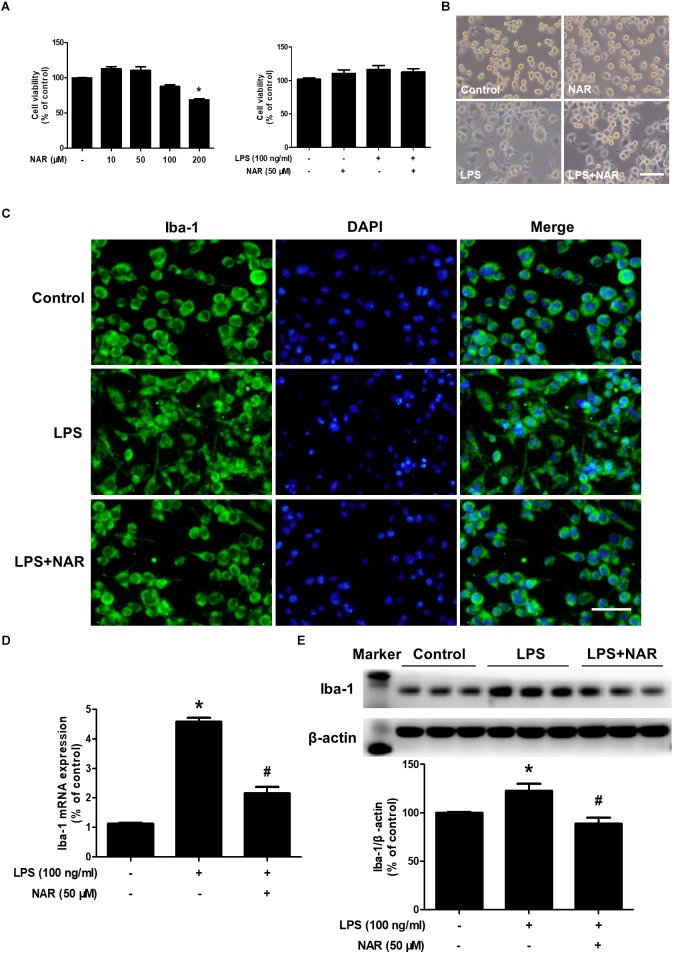
NAR attenuated LPS-induced microglial activation. BV-2 cells were treated with different concentrations of NAR for 24 h, cell viability was measured by MTT assay **(A)**. In addition, BV-2 cells were pretreated with NAR (50 μM) for 1 h and then incubated with LPS (100 ng/ml) for 24 h, cell viability were determined by MTT assay **(A)** and cell morphology was observed via an optical microscopeand **(B)**. Microglial activation was visualized by immunostaining with an anti-Iba-1 antibody **(C)**. Activation of microglia was quantitated by RT-PCR **(D)** and western blot analysis **(E)**. The ratio of densitometry values of Iba1 with β-actin was analyzed and normalized to each respective control cultures. Results were the mean ± SEM from three independent experiments performed in triplicate. ^∗^*p* < 0.05 compared to the control cultures. ^#^*p* < 0.05 compared to LPS-treated cultures. Scale bar = 50 μm.

As shown in Figures 1A,B, MTT assay indicated that NAR (100 μM) decreased BV2 cell viability and had cytotoxicity up to the concentration of 200 μM.BV-2 cells were exposed to NAR (50 μM) and LPS (100 ng/ml) for 24 h, cell morphology and cell viability were first determined. In addition, both NAR (50 μM) and LPS (100 ng/ml) had no significant toxic effects on the cell viability. Meanwhile, LPS and NAR had no effect on BV2 cells proliferation (Supplementary Figure S1). To discern the effects of NAR on LPS-induced microglial activation, the morphological changes were evaluated via immunostaining using an anti-Iba-1 (a specific microglial marker) antibody. As indicated in Figure 1C, in LPS-treated cultures, activated microglia illustrated irregular shapes and amoeboid status. However, NAR attenuated LPS-induced morphological changes of microglia with exhibiting resting round and small cells. In addition, western blot analysis provided quantitative estimation of microglial activation. As shown in Figures 1D,E, NAR inhibited LPS-induced increase of Iba-1 mRNA level and protein expression.

### NAR Switched Microglial M1 to M2 Polarization

**FIGURE 2:**
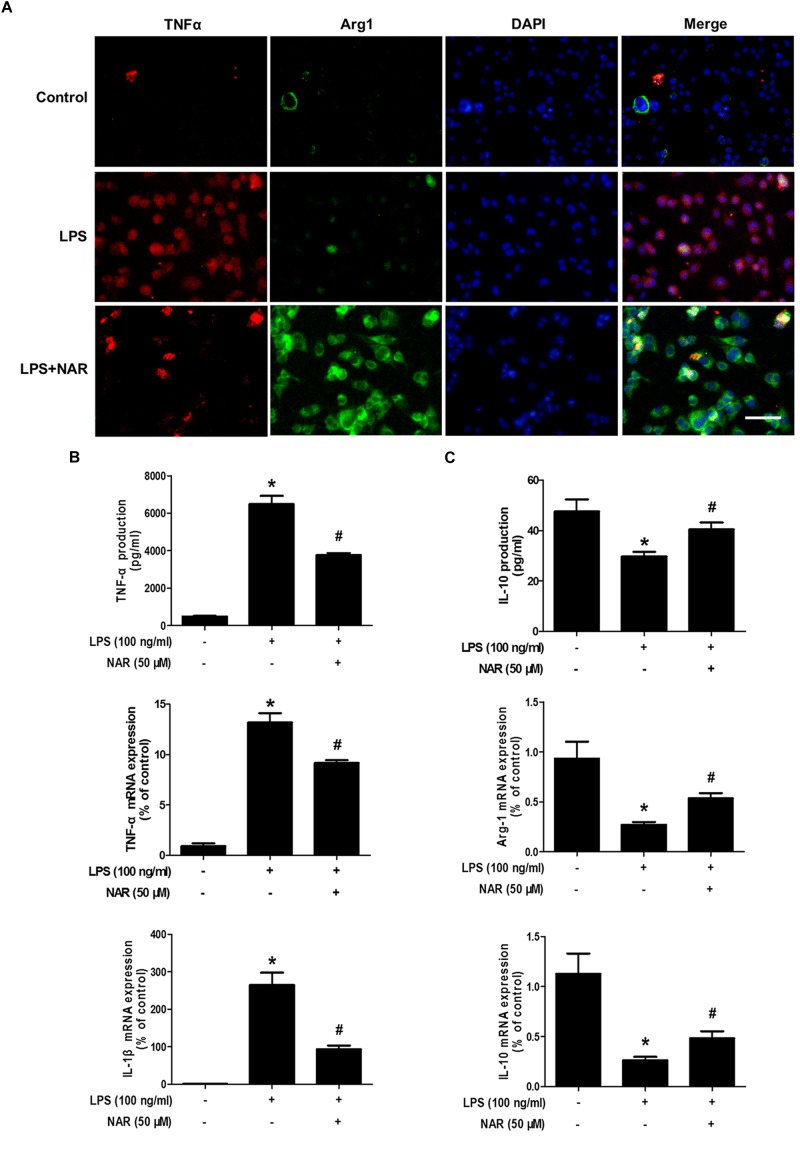
NAR switched microglial M1 to M2 polarization. BV-2 cells were pretreated with NAR (50 μM) for 1 h followed by LPS (100 ng/ml) adminstration for 24 h. Cultures were visualized by immunostaining with anti-TNF-α and Arg-1 antibodies **(A)**. The levels of TNF-α and IL-10 in supernatant were detected by ELISA and the whole cells were collected to detect the gene expressions of TNF-α, IL-1β, Arg-1, and IL-10 by real time RT-PCR **(B,C)**. Results were the mean ± SEM from three independent experiments performed in triplicate. ^∗^*p* < 0.05 compared to the control cultures. ^#^*p* < 0.05 compared to LPS-treated cultures. Scale bar = 50 μm.

It is well known that TNF-α and IL-1β were used as the marker of M1 polarization, whereas Arg-1 and IL-10 was applied as the marker of M2 polarization. The above observations prompted us to explore whether NAR directly switches microglial M1 to M2 phenotype. As shown in Figure 2A, less TNF-α and Arg-1 immunoreactivity was detected in control cultures. Notably, strong TNF-α immunoreactivity and less Arg-1 immunoreactivity were indicated in LPS-treated cultures. After NAR treatment, TNF-α immunoreactivity was decreased, and Arg-1 immunoreactivity was increased. As expected, the increased mRNA and extracellular protein expressions of TNF-α and IL-1β were observed in LPS-treated cultures. NAR-treated microglia expressed higher levels of mRNA and extracellular protein of anti-inflammatory cytokine Arg-1 and IL-10 than those exposed to LPS shown in Figures 2B,C. In addition, NAR alone had no significant effect on M1/M2 polarization (Supplementary Figure S2).

### NAR Inhibited MAPK Signaling Pathway Activation

**FIGURE 3:**
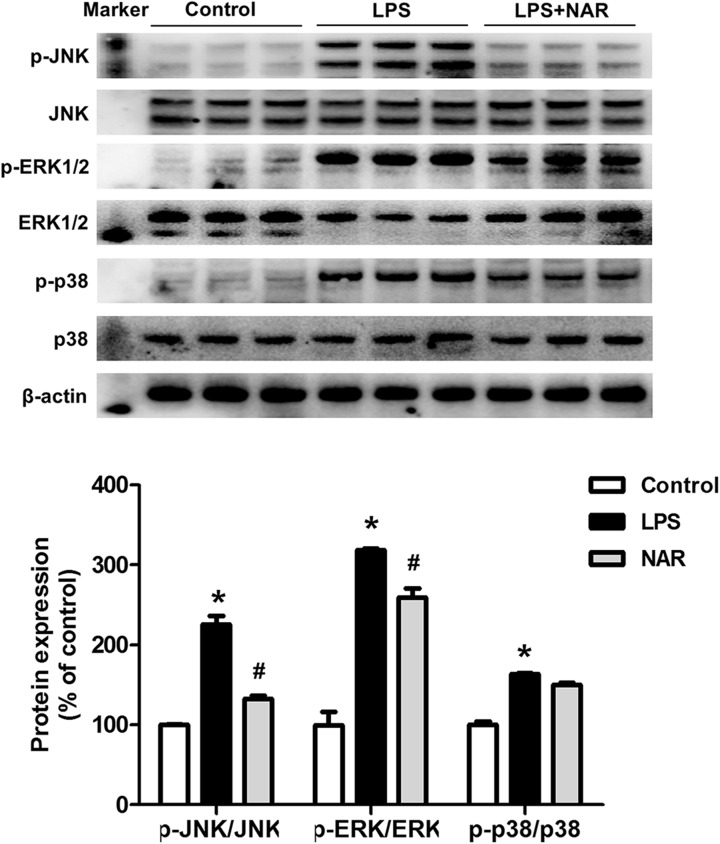
NAR inhibited MAPK signaling pathway activation. BV-2 cells were treated with NAR (50 μM) for 1 h prior to LPS (100 ng/ml) treatment. After LPS (100 ng/ml) stimulation for 24 h, cultures were harvested to detect the protein expressions of JNK, p-JNK, ERK1/2, p-ERK1/2, p38, p-p38 by western blot assay. The ratio of densitometry values of p-ERK1/2, p-JNK and p-p38 with total ERK1/2, JNK and p38 was assessed and normalized to each respective control group. Results were the mean ± SEM from three independent experiments performed in triplicate. ^∗^*p* < 0.05 compared to the control cultures. ^#^*p* < 0.05 compared to LPS-treated cultures.

To determine whether NAR could modulate the MAPK signaling pathway activation, the protein expressions of JNK, p-JNK, ERK1/2, p-ERK1/2, p38 and p-p38 were measured. As shown in Figure 3, NAR counteracted the LPS-induced phosphorylation of JNK and ERK1/2. Interestingly, NAR had the most obvious inhibitory effects on JNK activation. However, NAR didn’t show the recovery effects on LPS-induced activation of p38. Besides, NAR alone had no significant effect on MAPK signaling pathway (Supplementary Figure S2).

### NAR Promoted Microglial M1/M2 Polarization Through JNK Inactivation

**FIGURE 4:**
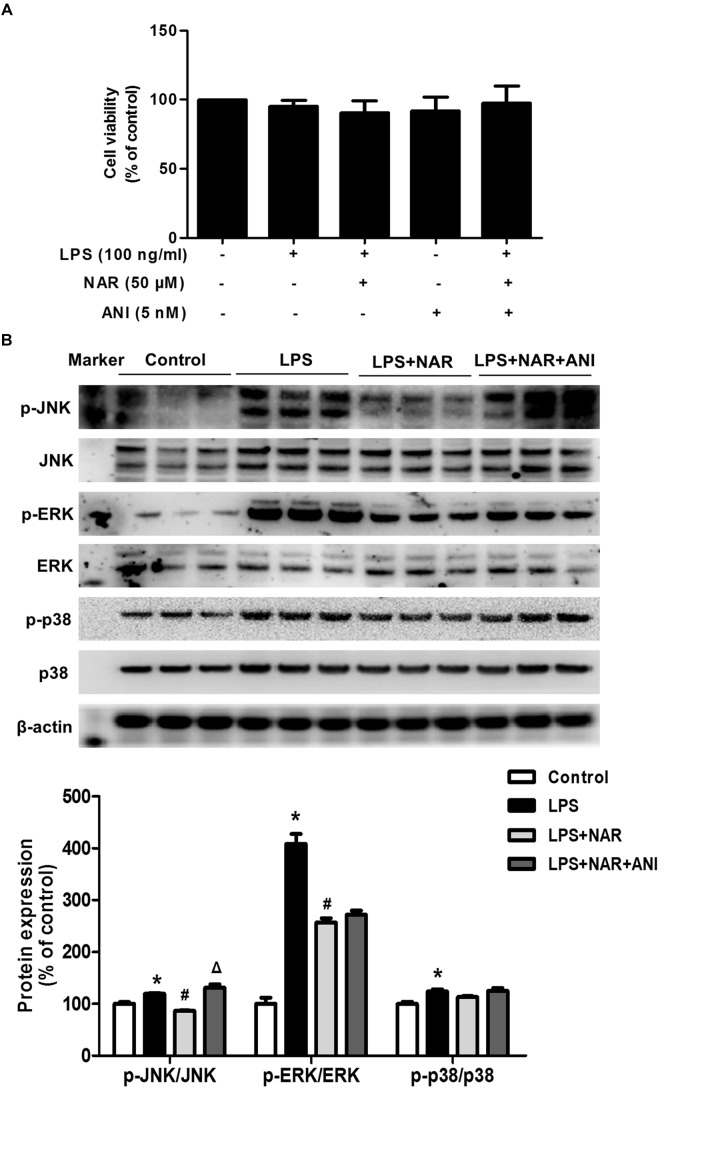
JNK activator attenuatedNAR-suppressed JNK activation. BV-2 cells were treated with NAR (50 μM) for 1 h and then ANI (5 nM) for 1 h followed by LPS (100 ng/ml) application for 24 h. Cell viability was determined by MTT assay **(A)**. The protein expressions of JNK, p-JNK, ERK1/2, p-ERK1/2, p38 and p-p38 were detected by western blot assay **(B)**. The ratio of densitometry values of p-ERK1/2, p-JNK and p-p38 with total ERK1/2, JNK and p38 was normalized to each respective control group. Results were the mean ± SEM from three independent experiments performed in triplicate. ^∗^*p* < 0.05 compared to the control cultures. ^#^*p* < 0.05 compared to LPS-treated cultures. ^#^*p* < 0.05 compared to LPS and NAR co-treated cultures.

Naringenin promoted microglia from M1 to M2 polarization and inhibited JNK and ERK1/2 phosphorylation, especially JNK phosphorylation. However, the specific target mediating this action was still unclear. Anisomycin (ANI), a selective agonist of JNK, was thus used to verify the role of JNK activation in NAR-mediated microglial M1/M2 polarization. First, as shown in Figure 4A, MTT assay indicated that ANI (5 nM) didn’t affect cell viability. In addition, ANI specifically counteracted NAR-elicited downregulation of phosphorylated-JNK but not phosphorylated-ERK1/2 and phosphorylated-p38 as shown in Figure 4B.

**FIGURE 5:**
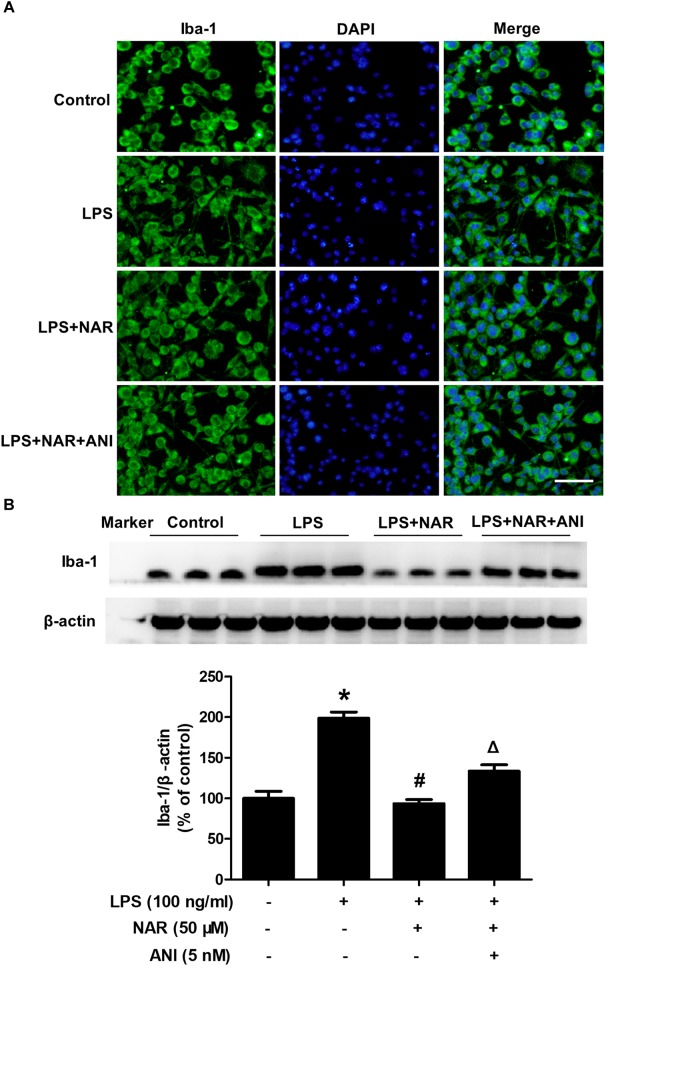
NAR inhibited microglia activation through JNK inactivation. BV-2 cells were treated with ANI (5 nM) for 1h after NAR pretreatment for 1 h. Then, LPS (100 ng/ml) were added into cultures. After LPS stimulation for 24 h, microglial activation was visualized by immunostaining **(A)** and quantitated by western blot analysis **(B)** using an anti-Iba-1 antibody. The ratio of densitometry values of Iba1 with β-actin was analyzed and normalized to each respective control cultures. ^∗^*p* < 0.05 compared to the control cultures. ^#^*p* < 0.05 compared to LPS-treated cultures. ^#^*p* < 0.05 compared to LPS and NAR co-treated cultures. Scale bar = 50 μm.

Next, we assessed and compared the functional recovery between NAR-treated cultures and NAR-ANI co-treated cultures to further determine the role of JNK on NAR-suppressed microglial activation. As shown in Figure 5A, NAR and ANI co-treated cultures exhibited irregular shapes and amoeboid status, parallel to the morphological changes in LPS-treated cultures. Western blot analysis further indicated that NAR-inhibited microglia activation was abrogated by ANI treatment shown in Figure 5B. These results pointed out a critical role of the activation of JNK in NAR-inhibited microglial activation.

**FIGURE 6:**
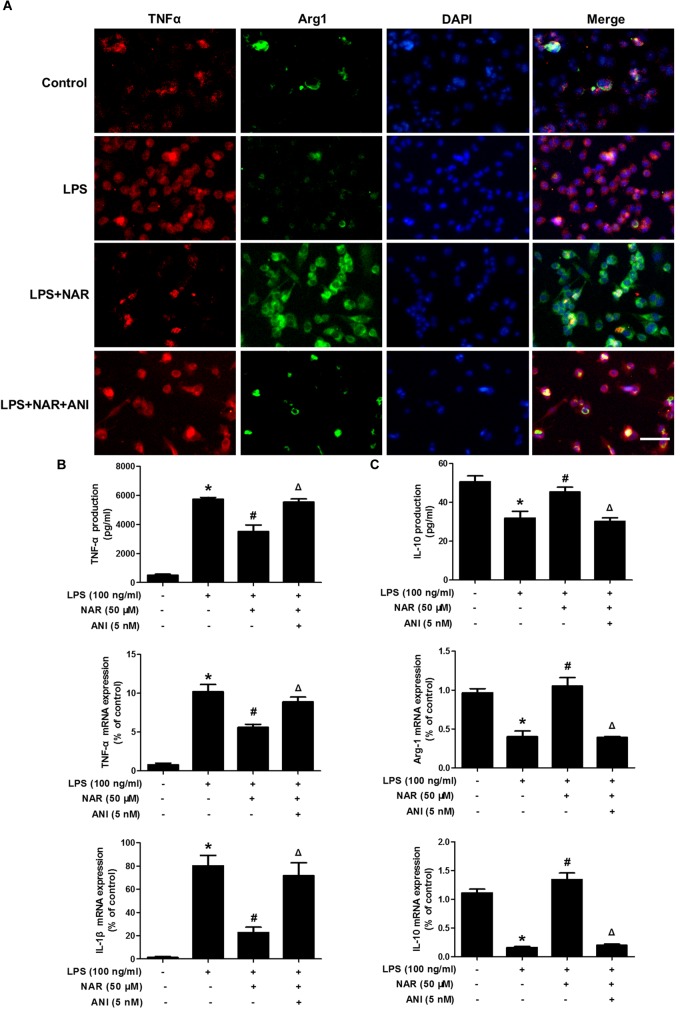
NAR promoted microglial M1/M2 polarization via the inhibition of JNK activation. BV-2 cells were treated with NAR (50 μM) for 1 h and ANI (5 nM) for another 1 h and then stimulated by LPS (100 ng/ml) for 24 h. Cultures were immunostained with anti-TNF-α and anti-Arg-1 antibodies **(A)**. Culture medium was collected to detect the contents of TNF-α and IL-10 by ELISA and the whole cells were collected to measure the gene expressions of TNF-α, IL-1β, Arg-1, and IL-10 by real time RT-PCR **(B,C)**. Results were the mean ± SEM from three independent experiments performed in triplicate. ^∗^*p* < 0.05 compared to the control cultures. ^#^*p* < 0.05 compared to LPS-treated cultures. ^#^*p* < 0.05 compared to LPS and NAR co-treated cultures. Scale bar = 50 μm.

Further studies were then conducted to evaluate the role of JNK on microglia M1/M2 polarization. As shown in Figure 6A, NAR and ANI co-treated cultures exhibited strong TNF-α (M1) immunoreactivity and less Arg-1(M2) immunoreactivity compared with NAR-treated culture. Furtherly, NAR-mediated reversal of LPS-induced downregulation of the M2 marker (Arg-1 and IL-10) and upregulation of the M1 marker (TNF-α and IL-1β) was abrogated by ANI administration as shown in Figures 6B,C. Thus, NAR switched microglial M1 to M2 polarization via a JNK-dependent pathway.

## Discussion

The current study indicated that NAR shifted the M1 pro-inflammatory microglia phenotype to the M2 anti-inflammatory M2 microglia state, thus inhibiting microglia-mediated neuroinflammation. In addition, the effects of NAR on microglial polarization was dependent on MAPK signaling, particularly JNK inactivation, as evidenced by the fact that the selective activator of JNK abolished NAR-promoted M2 polarization and further NAR-inhibited microglial activation. Together, this study demonstrated that NAR promoted microglia M1/M2 polarization, thus conferring anti-neuroinflammatory effects via MAPK-dependent inactivation.

To date, oxidative stress, mitochondrial dysfunction and environmental exposure have been characterized to be closely associated with the pathogenesis of neurological disorders. However, the underlying mechanisms remain unelucidated. Growing evidence has confirmed that neuroinflammation is involved in the pathogenesis of neurological disorders. The hallmark of neuroinflammation is the glial activation, especially microglial activation (Tentillier et al., 2016). Once activated by brain injury or inflammogen, microglia could release various types of pro-inflammatory and cytotoxic factors. The accumulation of these neurotoxic factors contributed to the surrounding neuronal damage. However, the continuous dying of neurons, in turn induced the secondary activation of microglia and the activated microglia further elicited neuronal damage (Block et al., 2007). Taken together, a vicious cycle leading to the prolonged neuroinflammation and the progressive neuronal loss was created (Gao and Hong, 2008). Thus, inhibition of microglial activation-mediated neuroinflammation might be promising therapeutic strategy for neuroprotection. The present study indicated that NAR inhibited microglia activation-mediated neuroinflammation. These results were consistent with the previous studies that NAR alleviated neuropathic pain through inhibiting microglia-induced neuroinflammation (Hu and Zhao, 2014).

In addition, a great number of studies set out to consider that microglia were highly plastic cells that could assume two diverse phenotypes and participate in different functional programs in response to the pathology of CNS. Recently, similar to the classical M1 phenotype versus the alternative M2 phenotype paradigm defined for macrophages, microglial M1/ M2 polarization has been coined and recognized in various neurological disorders, such as traumatic brain injury, stroke, and neurodegenerative diseases (Wang et al., 2013; Wu et al., 2016; Cheon et al., 2017). Typically, the activated M1 phenotype microglia produce various destructive pro- inflammatory factors that result in neuronal damage. As one of the most interesting M1 microglial polarization markers, TNF-α has been confirmed to be implicated in the pathology of neurological disorders (Makuch et al., 2013). IL-1β could be released by activated M1 microglia and the intrathecal administration of IL-1β was discerned to exert algesic actions (Kawasaki et al., 2008). In contrast, the alternatively activated M2 phenotype microglia generate numerous protective and neurotrophic factors and then underly the neuroprotective properties (Prinz and Priller, 2014). Therefore, the pro- and anti-inflammatory responses of microglia phenotype need to be balanced to prevent the potential detrimental activities of an uncontrolled and prolonged inflammation. So far, several lines of evidence presented for the role of neuroinflammation on neurodegenerative diseases pointed to a prolonged and uncontrolled activated microglia M1 state which led to additional continuous neuronal damage (Mantovani et al., 2013). Nevertheless, based on previous non-steroidal anti-inflammatory drug and Alzheimer’s disease anti-inflammatory prevention trial studies, simply suppressing inflammation via inhibiting M1 activation would likely not exert overall benefits (Becker et al., 2011). On the contrary, promoting the shift of microglial M1 to M2 phenotype while inhibiting microglia M1 state has been emerged as a more promising strategy for neuroinflammation-related disorders treatment (Li et al., 2018). Currently, most of the compounds suppressed neuroinflammation via simply inhibiting microglial M1 phenotype. However, few compounds were verified to promote microglial polarization to the M2 phenotype (Huang et al., 2017). Since NAR attenuated neuroinflammatory response, it is of significance to investigate the role of NAR on the modulation of microglial polarization. This study indicated that NAR robustly inhibited microglial M1 phenotype markers expressions and promoted microglia polarization toward the M2 anti-inflammatory phenotype, which might contribute to NAR-mediated neuroprotective actions against neuroinflammation.

Further analysis of cascade signaling events underlying NAR-mediated microglial polarization demonstrates the involvement of mitogen-activated protein kinases (MAPK) signaling pathway. MAPK pathway is a highly conserved family of serine/threonine kinases, including ERK1/2, p38 and JNK isoforms. MAPK signaling not only participates in the regulation of inflammatory responses, but also promotes macrophage/microglia polarization into the M2 stage (Quero et al., 2017). For instance, activation of ERK1/2 and p38 was implicated in the regulation of pro-inflammatory factors production in activated microglia (Zhang et al., 2010a). Moreover, p38 played a crucial role in allowing Ecto-5′-nucleotidase to modulate microglial M1/M2 polarization (Xu et al., 2018). In addition, a common involvement of JNK has also been studied in the regulation of both microglial iNOS and IL-1β expressions (Zhang et al., 2010b). Also, JNK was involved in exosomes-triggered macrophage polarization (Xiao et al., 2018). Thus, MAPK signaling is considered as an attractive target for inflammatory diseases treatment. Recently, some synthetic and natural compounds that could activate MAPK to exert anti-inflammatory effects and promote microglial M2 polarization (Bhatia et al., 2016; Xiang et al., 2018). Therefore, it was of interest to investigate the relationship between NAR-mediated anti-neuroinflammatory actions and MAPK activation. In the present study, we first found that NAR suppressed the phosphorylation of JNK and ERK1/2 in BV-2 cells, while p38 was not altered by NAR after LPS stimulation. Next, we tested whether NAR-mediated microglia polarization toward M2 state was dependent on JNK inactivation. Of note, the specific JNK activator abolished NAR-promoted microglia polarization further inhibiting microglial activation. The current data suggested that NAR-mediated microglial M1/M2 polarization was dependent on the inactivation of MAPK signaling.

In summary, this study demonstrated that NAR administration inhibited the microglial M1 phenotype and shifted the microglial polarization toward M2 state via the inhibition of MAPK signaling activation, which was accompanied by the reduced neuroinflammatory response. The current results supported the potential pharmaceutical application of NAR in neuroinflammation-related neurological disorders therapies.

## Conclusion

This study illustrates that NAR promoted microglial polarization toward the M2 phenotype through MAPK-dependent inactivation. These findings provide a new evidence that NAR might have considerable value as a potent agent for neuroinflammatory diseases treatment.

## Author Contributions

FZ conceived and designed theexperiments. BZ, G-QW, and Y-ZW participated in the experiments performance. G-QW, D-DL, J-SS, and FZ finished the data analysis. FZ and G-QW wrote, revised, and checked the article. All authors have reviewed the contents of the manuscript, validated the accuracy of the data, and approved the submitted manuscript.

## Conflict of Interest Statement

The authors declare that the research was conducted in the absence of any commercial or financial relationships that could be construed as a potential conflict of interest.

## Supplementary Material

FIGURE S1 The effects of NAR on cell proliferation. BV-2 cells were treated with NAR (50 μM) for 1 h prior to LPS (100 ng/ml) treatment. After LPS (100 ng/ml) stimulation for 24 h, cultures were harvested to detect the protein expressions of PCNA by western blot assay.

FIGURE S2 The effects of NAR alone on M1/M2 polarization and MAPK signaling pathway. BV-2 cells were treated with NAR (50 μM) for 24 h. The gene expressions of TNF-α, IL-1β, Arg-1, and IL-10 were detected by real time RT-PCR. The protein expressions of JNK, p-JNK, ERK1/2, p-ERK1/2, p38, p-p38 were measured by western blot assay.
