# Supplementary figures and images for: Targeting MAPK Pathways by Naringenin Modulates Microglia M1/M2 Polarization in Lipopolysaccharide-Stimulated Cultures
Source: Front Cell Neurosci. 2019 Jan 11;12:531. doi: 10.3389/fncel.2018.00531 (PMC6336899; doi:10.3389/fncel.2018.00531)

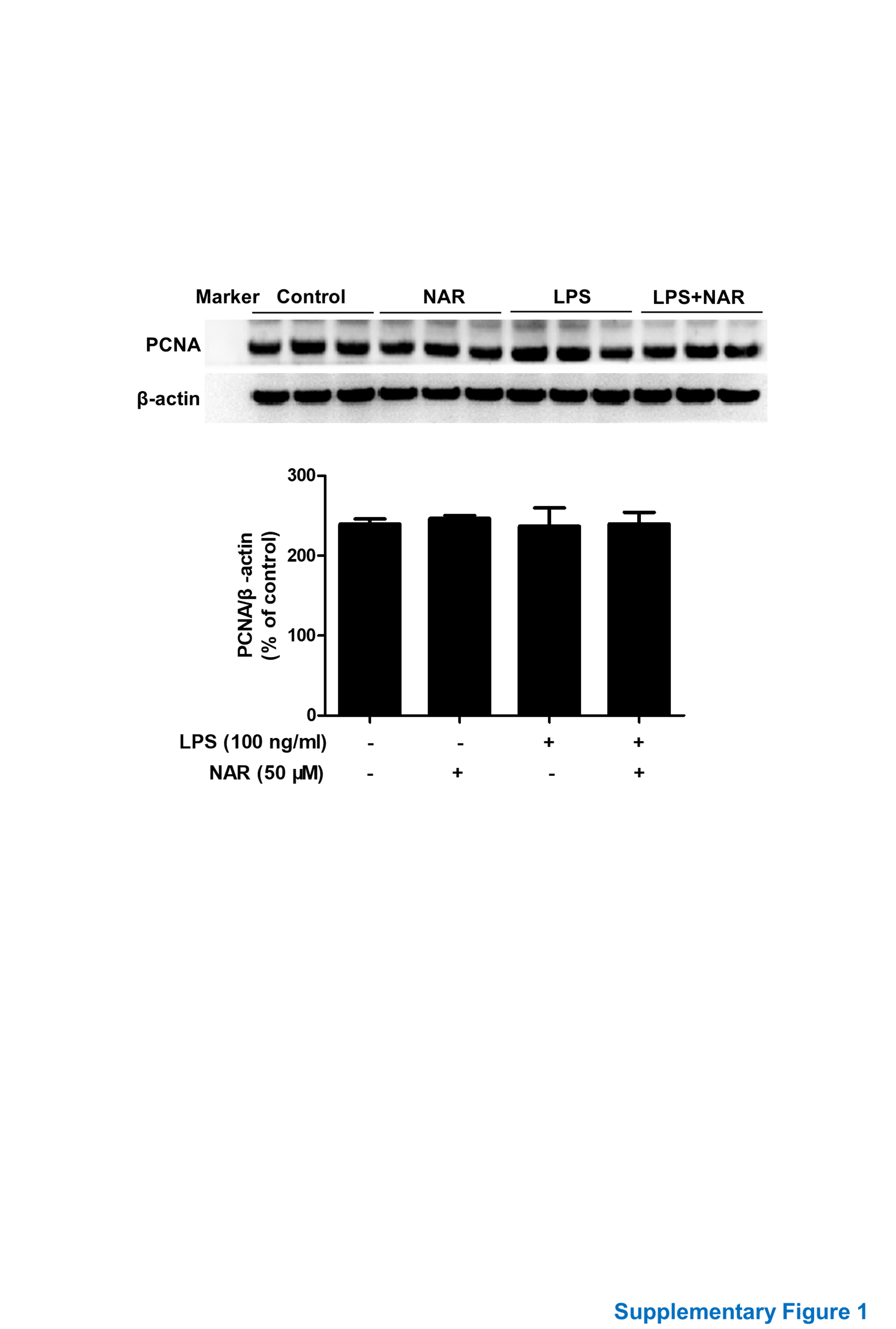

Supplement: FIGURE S1 — The effects of NAR on cell proliferation. BV-2 cells were treated with NAR (50 μM) for 1 h prior to LPS (100 ng/ml) treatment. After LPS (100 ng/ml) stimulation for 24 h, cultures were harvested to detect the protein expressions of PCNA by western blot assay. [file Image_1.TIF]

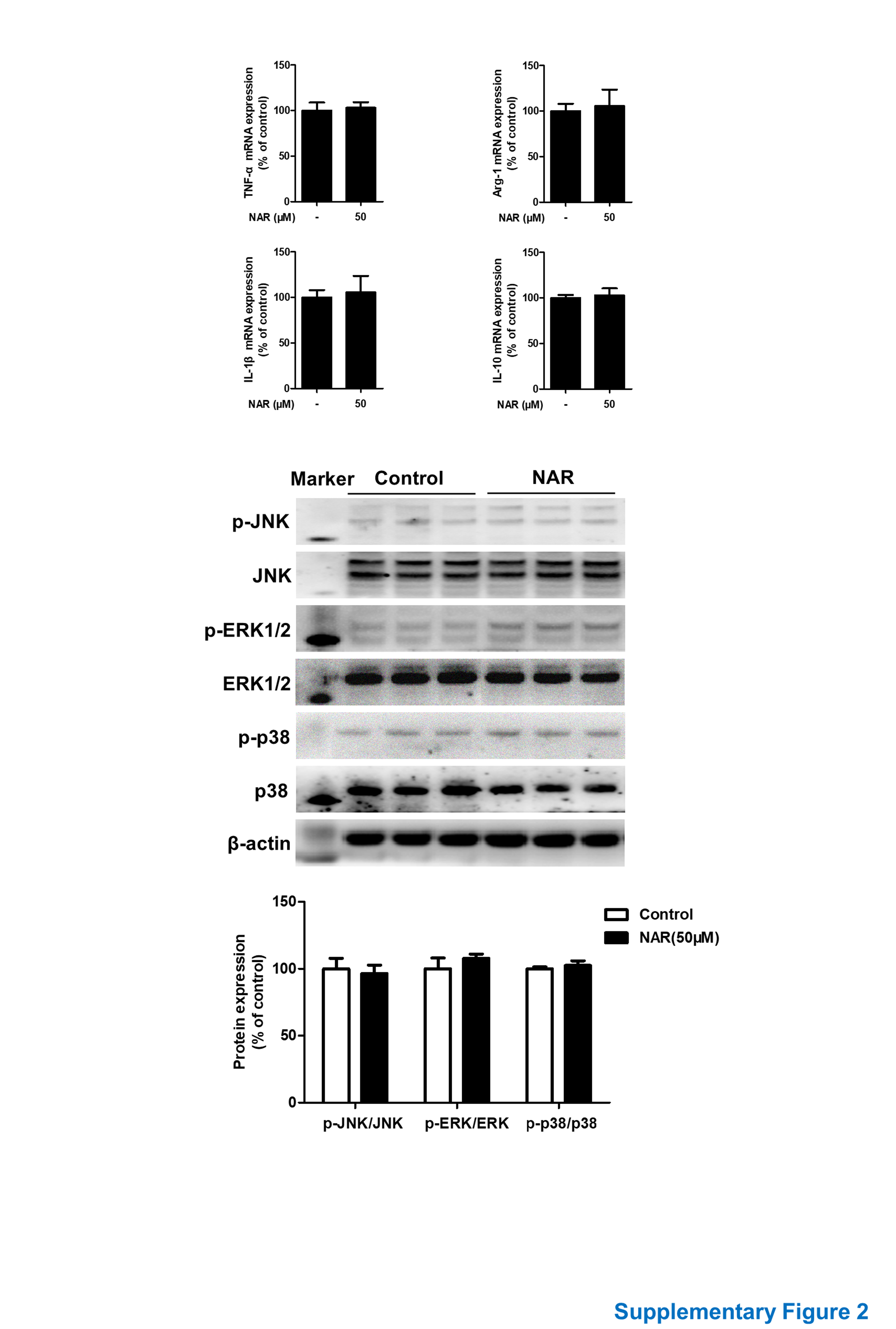

Supplement: FIGURE S2 — The effects of NAR alone on M1/M2 polarization and MAPK signaling pathway. BV-2 cells were treated with NAR (50 μM) for 24 h. The gene expressions of TNF-α, IL-1β, Arg-1, and IL-10 were detected by real time RT-PCR. The protein expressions of JNK, p-JNK, ERK1/2, p-ERK1/2, p38, p-p38 were measured by western blot assay. [file Image_2.TIF]
